# Capturing Movement Behaviors in Latinas: Feasibility, Validity, and Acceptability Study of an Ecological Momentary Assessment Protocol

**DOI:** 10.2196/75855

**Published:** 2025-11-05

**Authors:** Jaclyn P Maher, Peyton A Greco, Eugenia Camacho Fernandez, Brynn L Hudgins, Sandra E Echeverria

**Affiliations:** 1 Department of Kinesiology, University of North Carolina at Greensboro, 1408 Walker Ave, Greensboro, NC, 27412, United States, 1 3362561379; 2 Department of Public Health Education, University of North Carolina at Greensboro, Greensboro, NC, United States

**Keywords:** experience sampling, ambulatory assessment, Hispanic, physical activity, exercise, sitting, accelerometer, women

## Abstract

**Background:**

Latinas are one of the largest and fastest-growing female ethnic groups in the United States and have high levels of physical inactivity and sedentary behavior (SB), contributing to a disproportionate burden of chronic health conditions. An ecological momentary assessment (EMA) involves the use of smartphone-based data collected in real time to assess health behaviors and outcomes.

**Objective:**

We examined the feasibility, validity, and acceptability of an EMA protocol assessing physical activity (PA) and SB in Latina adults.

**Methods:**

For 7 days, 67 Latinas (average age 39 years, SD = 13.6; n=37, 55.2% earning less than US $50,000/year; n=53, 79.1% foreign-born; and n=49, 73.1% of Mexican or Mexican American origin) completed a signal-contingent EMA protocol with 3 prompts per day and wore an ActiGraph GT3X accelerometer to measure levels of PA and SB. EMA prompts inquired about current behavior, feelings, beliefs, social conditions, and contexts.

**Results:**

Latinas completed 69.7% (892/1279) of EMA prompts. They were more likely to respond to EMA prompts when engaged in more SB (odds ratio [OR] 1.04, 95% CI 1.01-1.06) and less light-intensity PA (OR 0.97, 95% CI 0.94-0.99) in the 30 minutes around the prompt. Accelerometer data validated self-reported occasions of PA and SB via EMA. The majority of participants (>70%) were satisfied with the protocol and expressed interest in participating in future studies.

**Conclusions:**

EMA is a feasible, valid, and acceptable methodology for capturing movement behaviors among Latinas, which can provide insights into the antecedents and consequences of these behaviors in their daily lives.

## Introduction

Latina or Hispanic adult women (hereafter, “Latinas”) are one of the largest, fastest-growing female ethnic groups in the United States and engage in lower levels of physical activity (PA) compared to Latino men and non-Latina women [1,2]. Recent estimates suggest that one-third of Latinas in the United States report no moderate-to-vigorous-intensity physical activity (MVPA; outside of work), the highest of any racial or ethnic group [3]. Conversely, Latinas tend to engage in more light-intensity physical activity (LPA) and less sedentary behavior (SB) compared to their White and Black counterparts [4,5]. The unique movement behavior profiles among Latinas likely result from unique barriers and facilitators that Latinas encounter in their daily lives. Yet, our understanding of Latinas’ engagement in movement behaviors (ie, MVPA, LPA, and SB) and determinants of those behaviors in naturalistic settings is limited, hampering efforts to effectively intervene [6]. Therefore, it is not surprising that Latinas in the United States experience disproportionately high rates of chronic conditions closely linked to low levels of MVPA such as diabetes, hypertension, obesity, and certain cancers.

The ecological momentary assessment (EMA) has increased in popularity over the last 15 years in PA research as a methodological approach to better capture health behaviors and their determinants as they occur in daily life [7]. Specifically, EMA is a real-time data capture methodology designed to intensively and repeatedly assess participants’ behaviors, experiences, feelings, and contexts surrounding a phenomenon of interest [8]. Such an approach can improve ecological validity and reduce recall errors [9], ultimately improving our understanding of when, where, with whom, and why movement behaviors occur.

Despite the potential advantages of this methodological approach, no studies have explicitly evaluated the feasibility, validity, and acceptability of EMA to capture movement behaviors in Latinas. Latinas are likely to face unique barriers to participation in research involving methodologies such as EMA given documented issues related to lack of trust in research, limited rapport with research staff, and cultural insensitivity of research to the unique values, traditions, and beliefs of the Latino/a community [10,11]. Other issues associated with broader research participation among Latinas include concerns about immigration or undocumented status, lack of access to Spanish-speaking staff or interpreters, and lack of childcare [10,11].

Therefore, there is a need to establish the feasibility, validity, and acceptability of a smartphone-based EMA study to assess movement behaviors in Latinas. In this study, we examined the feasibility, validity, and acceptability of a 7-day EMA protocol assessing PA and SB. To determine feasibility, we examined rates of compliance with elements of the study protocol (ie, EMA, accelerometer wear time). EMA studies across a variety of designs, samples, and research fields have reported an average compliance with EMA protocols of 79% [12]. However, given that EMA studies focused on movement behaviors in other samples of minoritized women have reported lower compliance [13] balanced with the need to capture representative and ecologically valid data on participants’ daily experiences, we deemed a compliance rate of 70% to be acceptable. We also examined measurement reactivity to determine whether accelerometer-derived behavioral data were affected or changed because of answering the EMA prompts. With respect to validity, we investigated the degree to which EMA-reported behaviors corresponded with the amount of MVPA, LPA, and SB captured by the accelerometer over that same time. To our knowledge, we are not aware of any studies that have investigated compliance with, potential reactivity to, or validity of EMA reports with regard to LPA; however, given the unique movement behavior profiles of Latinas with high amounts of LPA and accumulating evidence regarding the health benefits of LPA [3-5,14], this is an important question to investigate. Finally, to establish acceptability, we used end-of-study feedback on specific elements of the study. This formative research is crucial for enhancing our understanding of the factors influencing movement behaviors and for developing personally tailored and context-sensitive movement-related interventions for Latinas.

## Methods

### Participants and Recruitment

Latinas living in Guilford County, North Carolina, and surrounding counties were recruited for participation in this study. Recruitment was done primarily through community centers serving predominantly Latina/o populations. Through conversations with community partners, effective methods for recruitment were identified. Print materials were placed at community locations. Further, announcements were made and recruitment tables with staff were present at specific community events (eg, English as a second language classes, soccer games, and family services). Community partners also communicated information about the study to organization patrons. Women were eligible if they were aged ≥18 years, self-identified as Latina or Hispanic, and had no limitations that would prevent engaging in PA. If women did not speak and read English or Spanish, were pregnant, or were unwilling to use their personal smartphone for the study, they were deemed ineligible to participate. We hired and trained bilingual, bicultural research staff to recruit, enroll, and check in with participants over the course of the study.

### Procedures

Following eligibility screening, Latinas attended the introductory training session (day 1) at one of three community locations. At the training session, a research staff member provided a broad overview of the study (eg, procedures, risks or benefits, and compensation). After the overview, individuals were asked to review the consent form (written in English) and sign to indicate their willingness to participate in the study. Research staff indicated that they could read the consent form to participants, if desired. Although we did not collect specific records of whether participants read the consent themselves or had the staff member read it to them, based on anecdotal staff reports, most participants read the consent form themselves. Research staff were available to answer any questions individuals had. Once participants consented, they completed a baseline questionnaire to provide basic demographic and health information. Participants were also familiarized with an accelerometer, which was to be worn on the hip during all waking hours unless they were showering, bathing, or swimming. Research staff also provided more details on the EMA study procedures during the training session and assisted participants in downloading the smartphone app, LifeData, onto their personal smartphones. The app delivered the signal-contingent EMA protocol. The EMA protocol delivered a questionnaire at a random time within three 2-hour windows (ie, morning: 6:30-8:30 AM; afternoon: 12-2 PM; evening: 6-8 PM). The EMA surveys took 2‐3 minutes to complete, and participants could receive between 29 and 35 items at each prompt depending on participant responses at the time of the prompt (eg, if they were at work and if they were alone). Screenshots of all EMA items can be found in Figure S1 in Multimedia Appendix 1. When reviewing the EMA items during the training sessions, as well as at the beginning of each EMA prompt, participants were presented with definitions of key constructs to help orient participants to what was meant by specific terms that appeared in the questions. For instance, participants were provided a definition of physical activity that aligned with MVPA (eg, moderate to hard physical effort and breathing harder than normal). The EMA protocol was available in English and Spanish and continued for 7 consecutive days.

After the delivery of an EMA prompt, participants had 30 minutes to answer the EMA. After 30 minutes, if the EMA prompt was still unanswered, the questionnaire became inaccessible and was counted as missing. Research staff monitored compliance in real time and contacted all participants 2‐3 days into the EMA protocol to check in and address any issues related to questionnaire delivery, compliance, etc.

Following the 7-day study period, participants returned the study equipment at a community location and were compensated for their time. Community locations were also compensated for granting the research team space to meet with participants. Finally, participants were asked to complete a brief questionnaire to gather feedback about their experience in the study.

### Measures

#### EMA-Reported Physical Activity and Sedentary Behavior

At each EMA prompt, participants were asked “What were you doing just before the phone went off?” and were given the option to select all responses that were applicable (eg, watching television or movies; using a computer, tablet, or phone; physical activity or exercise; running errands; and household chores [15-17]; see Multimedia Appendix 1 for a complete list of response options). Given our interest to determine the validity between self-reported PA and device-measured PA, occasions in which participants selected “physical activity/exercise” at the time of the prompt were classified as currently engaged in PA at that occasion. Responses were coded so that occasions in which PA was not reported were the reference (coded as 0) and occasions in which PA was reported were the comparison (coded as 1). Aside from occasions where participants reported engaging in “physical activity/exercise,” they were asked if they were sitting while doing their current activity. We classified participants as engaging in SB at the time of the prompt if they reported sitting while doing their current activity. Responses were coded so that occasions where sitting was not reported were the reference (coded as 0) and occasions where sitting was reported were the comparison (coded as 1). Regardless of whether participants indicated they were currently engaged in PA or SB, they were not asked to report the duration of their current PA or SB.

#### Device-Based Physical Activity and Sedentary Behavior

The ActiGraph GT3X-BT accelerometer measured participants’ PA and SB. Data from this device were downloaded in 30-second epochs, and movement behaviors were operationalized as minutes of MVPA (ie, ≥2020 counts/minute), LPA (ie, 100‐2019 counts/minute), and SB (<100 counts/minute) [18,19]. Time stamps from the device and EMA questionnaires were used to pair the 2 data sources and create windows of accelerometer data before, after, or around the EMA prompt. Movement behavior data accumulated in the 15 minutes before, 15 minutes after, or 30-minute window around (ie, ±15 minutes) the EMA prompt was used in analyses. Nonwear time was determined based on consecutive minutes of 0 activity counts [19]. Time intervals in which valid wear time amounted to at least half of the interval were considered valid and included in the analysis (eg, ≥7.5 minutes of wear in the 15 minutes before the prompt).

#### Time

We created variables representing the time of day and day of the week EMA prompts were delivered. EMA prompts delivered between 6:30 and 8:30 AM were classified as morning prompts, 12 and 2 PM as afternoon prompts, and 6 and 8 PM as evening prompts. Prompts delivered from Monday to Friday were classified as weekday prompts, and prompts delivered on Saturday and Sunday were classified as weekend prompts. Finally, an EMA prompt number variable (ranging between 1 and 21, as this is the maximum number of prompts that could be delivered as part of the study) was created to account for time in study.

#### Demographic, Family, Work, Social, and Structural Determinants

During the introductory training session, participants completed a baseline questionnaire that assessed demographic and health information including age, nativity, and self-reported health. Although not relevant to the aims of this manuscript, at each EMA prompt, participants reported on family, work, and social conditions and potential stressors they were experiencing. Latinas’ geographic location at the time of the prompt was also recorded at each EMA prompt.

#### Study Feedback

At the end of the study, participants provided their perceptions of study procedures, their overall experience, and their willingness to complete smartphone-based or accelerometer-related study procedures in future studies. Items and response options are provided in the Results section.

### Data Analysis

Our analytic approach was informed by previous work investigating the feasibility and validity of EMA in PA-related research in other populations [15-17]. As an intensive longitudinal study, we fit a series of multilevel models to account for the nested structure of our data (ie, observations nested within people) to investigate the aims previously defined [20,21]. Descriptive statistics and frequencies were calculated to describe compliance (ie, total answered prompts completed divided by total delivered prompts).

To determine if engagement in movement behaviors influenced the likelihood of completing an EMA prompt, we fit a series of multilevel logistic regression models. More specifically, these models examined if engaging in movement behaviors (ie, MVPA, LPA, and SB in the 30-minute window [±15 minutes] around the EMA prompt) as well as temporal (ie, time of day, day of week, and time in study) or demographic factors (ie, age, preferred language, self-reported health, nativity status, and employment status) influenced whether or not an EMA prompt was completed. EMA prompts were dummy coded (ie, prompts completed were coded as 1; prompts not completed were coded as 0). Prompts were considered not completed if a participant did not open the EMA questionnaire within 30 minutes of delivery or the prompt was answered but the participant did not answer all questions included in the EMA questionnaire (ie, did not provide complete data at the EMA prompt).

The next set of models examined whether the time spent engaging in PA (or SB) in the 15-minute window before the EMA prompt differed from the time spent engaging in PA (or SB) in the 15-minute window after the EMA prompt. This is relevant as it provides insights into whether the EMA prompt resulted in any reactivity (or changes in PA or SB as a result of being prompted about their movement behaviors) [22,23]. We created a dummy-coded variable representing the 15 minutes prior (“window”=0) and after the EMA prompt (“window”=1) to examine its association with MVPA, LPA, and SB (one movement behavior per model).

Our final set of models examined criterion validity. We fit multilevel linear regression models to determine associations between EMA-reported PA and SB and accelerometer-derived PA and SB, respectively. We used EMA-reported PA (or SB) at the time of the prompt as the main predictor to determine associations with accelerometer-derived MVPA, LPA, or SB (one movement behavior per model). For all models in which MVPA was the outcome, model results were consistent regardless of using raw or transformed MVPA variables, so we opted to use raw MVPA values to aid in the interpretability of findings.

Based on a priori selection of covariates and prior studies, we adjusted all multilevel models for time and day of the prompt, time in study (ie, EMA prompt number), age (grand mean centered), employment status (dummy coded), language of questionnaire (dummy coded), nativity (dummy coded), and self-reported health (eg, [3,5,6,12,15,23-25]). Previous simulation studies indicate that having at least 50 units at the high order level (in our case, participants at level 2) with between 5 and 30 observations per unit (in our case, we averaged 10 valid observations per participant) is sufficient to generate accurate unstandardized coefficient and standard error estimates [26]. Quantitative analyses were conducted in Stata v. 18 and SAS v9.4.

Lastly, to establish acceptability, we evaluated reports of satisfaction with, convenience of, and disruptions due to study participation. In addition to Likert items assessing acceptability, participants were given the opportunity to provide additional feedback via an open-ended question. Only one-third (23/67, 34.3%) of the participants responded to the open-ended question, and responses were brief, with an average length of 10 words. Therefore, we did not conduct a formal qualitative analysis but highlighted quotes to represent common responses across respondents.

### Ethical Considerations

All study procedures were approved by the University of North Carolina at Greensboro’s institutional review board (IRB FY22-483). Written informed consent was obtained from all individual participants included in the study. Several measures were implemented to ensure participant privacy and confidentiality, including meeting with participants in private or secluded areas at community locations, using ID numbers to link participant data, and storing electronic or paper records in secure locations. Participants could earn up to US $50 in gift cards: US $20 for attending the introductory session and US $30 for completing the EMA and accelerometer protocol (not prorated based on compliance).

## Results

### Participant Characteristics and Completion of EMA Prompts and Accelerometer Wear

A total of 67 Latinas completed the study. Table 1 displays the demographic characteristics of study participants. Participants were, on average, 39 years of age (SD = 13.6). About half of the sample met physical activity guidelines and indicated they were from households earning less than US $50,000 per year. Approximately three-quarters (53/67, 79.1%) of the sample were foreign-born. Similarly, about three-quarters (49/67, 73.1%) of the sample were identified as Mexican or Mexican American.

**Table 1.** Demographic characteristics of study participants.

Participant characteristics	Values
Age (years) (n=67), n (%)	
18‐44	44 (65.7)
45‐64	21 (31.3)
≥65	2 (3.0)
Education (n=67), n (%)	
Some high school	18 (26.9)
High school or equivalent	23 (34.3)
Some college	12 (17.9)
College degree or postsecondary	12 (17.9)
Missing	2 (3.0)
Annual household income (US $) (n=67), n (%)	
<10,000	3 (4.5)
10,000‐24,999	11 (16.4)
25,000‐49,999	23 (34.3)
≥50,000	20 (29.9)
Do not know or not sure	10 (14.9)
Marriage status (n=67), n (%)	
Married or cohabitating	48 (71.6)
Single	12 (17.9)
Other	7 (10.5)
Ethnic group (n=67), n (%)	
Mexican or Mexican American	49 (73.1)
Central American	13 (19.4)
Other	5 (7.5)
Language spoken at home (n=67), n (%)	
Only Spanish	33 (49.2)
Mostly Spanish	15 (22.4)
Mostly English	2 (3.0)
Spanish and English equally	17 (25.4)
Self-reported health (n=67), n (%)	
Excellent	8 (11.9)
Very good	13 (19.4)
Good	27 (40.3)
Fair	19 (28.4)
Currently employed (n=67), n (%)	
Yes	41 (61.2)
Country of birth (n=67), n (%)	
United States	14 (20.9)
Mexico	40 (59.7)
Other	13 (19.4)
Physical activity (n=67), n (%)	
Meeting physical activity guidelines (based on average daily minutes of MVPA)^a^	37 (55.2)
Occasions engaging in physical activity or exercise (n=907), n (%)	
EMA-based^b^ report	35 (3.9)
Occasions engaging in sedentary behavior (n=901), n (%)	
EMA-based report	457 (50.7)
Moderate to vigorous physical activity (minutes), mean (SD)	
Device-based assessment (15 minutes prior to EMA prompt)	0.44 (1.42)
Light physical activity (minutes), mean (SD)	
Device-based assessment (15 minutes prior to EMA prompt)	5.01 (3.72)
Sedentary behavior (minutes), mean (SD)	
Device-based assessment (15 minutes prior to EMA prompt)	9.32 (4.21)

a MVPA: moderate to vigorous intensity physical activity.

b EMA: ecological momentary assessment.

There were 1279 EMA prompts delivered across all participants. Of the EMA prompts delivered, participants answered 927 (72.5%) EMA prompts and completed 892 (69.7%, range: 5%‐100%) EMA prompts. On approximately two-thirds of occasions, participants had valid actigraphy data in the 15 minutes before (n=842, 65.8%) or 15 minutes after (n=860, 67.2%) the EMA prompt. Frequencies and descriptive statistics for EMA-reported and device-based movement behaviors are displayed in Table 1.

### Factors Influencing Compliance With Study Procedures

Table 2 displays associations between demographic, temporal, and behavioral factors and compliance. There was no difference in odds of participants completing the EMA prompt (vs not) depending on the amount of MVPA engaged in in the 30-minute window around the EMA prompt (*P*>.05). However, Latinas were more likely to complete an EMA prompt if they engaged in more SB (odds ratio [OR] 1.04, 95% CI 1.01-1.06) and less LPA (OR 0.97, 95% CI 0.94-0.99) in the 30-minute window around the EMA prompt.

**Table 2.** Odds of completing an EMA^a^ prompt based on participant characteristics and current behaviors and temporal context.^b^

	Model 1: Multilevel logistic regression with MVPA^c^ as key movement behavior, OR^d^ (95% CI)	Model 2: Multilevel logistic regression with LPA^e^ as key movement behavior, OR (95% CI)	Model 3: Multilevel logistic regression with SB^f^ as key movement behavior, OR (95% CI)
Fixed effects			
Intercept	0.66 (0.17‐2.54)	0.84 (0.21‐3.35)	0.32 (0.08‐1.36)
Movement behavior (±15 minutes)	0.95 (0.90‐1.01)	0.97^g^ (0.94‐0.99)	1.04^g^ (1.01‐1.06)
Afternoon	1.83^g^ (1.08‐3.10)	1.85^g^ (1.09‐3.14)	1.74^g^ (1.02‐2.95)
Evening	2.51^g^ (1.47‐4.29)	2.36^g^ (1.38‐4.04)	2.22^g^ (1.29‐3.81)
Weekend day	0.66^g^ (0.43‐0.99)	0.67 (0.44‐1.02)	0.66^g^ (0.44‐0.99)
Notification number	1.08^g^ (1.04‐1.12)	1.08^g^ (1.04‐1.12)	1.08^g^ (1.04‐1.12)
Age	1.01 (0.97‐1.04)	1.01 (0.98‐1.04)	1.01 (0.98‐1.04)
EMA in Spanish	1.04 (0.38‐2.90)	1.07 (0.38‐3.00)	1.09 (0.39‐3.05)
Currently working	1.19 (0.57‐2.48)	1.21 (0.57‐2.54)	1.22 (0.59‐2.56)
US born	1.26 (0.38‐4.13)	1.23 (0.37‐4.09)	1.25 (0.38‐4.08)
Self-reported health	1.25 (0.85‐1.85)	1.27 (0.85‐1.88)	1.26 (0.85‐1.86)
Random effects			
Intercept	1.41 (0.78‐2.56)	1.45 (0.81‐2.63)	1.47 (0.77‐2.56)

a EMA: ecological momentary assessment.

b All models were based on 67 participants with 850 observations.

c MVPA: moderate to vigorous intensity physical activity.

d OR: odds ratio.

e LPA: light intensity physical activity.

f SB: sedentary behavior.

g *P*<.05.

Latinas were more likely to complete an EMA prompt in the afternoon, evening, and weekend compared to mornings and weekdays (except for the model with LPA predicting compliance). Participants were also more likely to complete an EMA prompt as participants received more notifications in the study (ie, time went on in the study). There was no difference in odds of completing an EMA prompt by between-person demographic factors (eg, age).

### Potential Reactivity to EMA Prompts

Latinas engaged in significantly more SB in the 15 minutes following the EMA prompt (*B*=0.49, SE 0.21, *P*=.02; mean 9.56, SD 4.14 minutes) compared to the 15 minutes before the prompt (mean 9.07, SD 4.34 minutes). However, there was no difference in MVPA (*B*=−0.04, SE 0.07; *P*=.54) or LPA (*B*=−0.3, SE 0.19; *P*=.11) in the 15 minutes before the EMA prompt compared to the 15 minutes after the EMA prompt (data not shown in table).

### Criterion Validity of EMA Responses

Table 3 displays results from models investigating the extent to which there was agreement between device-based movement behaviors and EMA reports of PA and SB. Latinas who self-reported engaging in PA at the time of the prompt averaged 3.6 more minutes of device-based MVPA (*B*=3.60, 95% CI 2.75‐4.45) and 2.32 more minutes of LPA (*B*=2.32, 95% CI 0.17-4.61) relative to occasions when Latinas did not report being physically active at the time of the prompt. Further, device-based assessments of SB corresponded with self-reported behavior via EMA. At the time of the prompt, Latinas averaged 4.11 more minutes of device-based SB in the 30-minute window (ie, ±15 minutes) on occasions when they reported sitting at the time of the prompt relative to occasions when they did not report sitting at the time of the prompt (*B*=4.11, 95% CI 3.10-5.11).

**Table 3.** Predicting minutes of movement behaviors in the 30-minute window (±15 minutes) around the EMA^a^ prompt to determine correspondence between EMA-reported and device-based behaviors.^b^

	Multilevel linear regression with MVPA^c^ as outcome, unstandardized coefficient (SE)	Multilevel linear regression with LPA^d^ as outcome, unstandardized coefficient (SE)	Multilevel linear regression with SB^e^ as outcome, unstandardized coefficient (SE)
Fixed effects			
Intercept	0.14 (0.42)	8.89^f^ (1.34)	17.09^f^ (1.47)
EMA-reported PA^g^	3.60^f^ (0.43)	2.32^f^ (1.17)	—^h^
EMA-reported SB	—^h^	—^h^	4.10^f^ (0.51)
Afternoon	–0.01 (0.28)	–0.86 (0.77)	1.84^f^ (0.82)
Evening	0.09 (0.28)	–1.31 (0.76)	2.14^f^ (0.82)
Weekend day	–0.25 (0.20)	–0.39 (0.52)	0.71 (0.57)
Notification number	0.01 (0.01)	0.02 (0.04)	–0.05 (0.05)
Age	–0.01 (0.01)	0.07^f^ (0.03)	–0.07^f^ (0.03)
EMA in Spanish	0.10 (0.27)	0.91 (0.92)	–1.29 (0.99)
Currently working	0.12 (0.19)	0.97 (0.67)	–0.57 (0.70)
US born	–0.01 (0.31)	–1.15 (1.05)	0.40 (1.13)
Self-reported health	0.14 (0.10)	0.25 (0.36)	–0.15 (0.38)
Random effects			
Intercept	0.03 (0.08)	2.46 (1.09)	2.77 (1.24)
Residual	4.90 (0.28)	34.67 (2.01)	40.35 (2.34)

a EMA: ecological momentary assessment.

b All models were based on 66 participants with 657 observations for models predicting MVPA and LPA, and 656 observations for the model predicting SB.

c MVPA: moderate to vigorous intensity physical activity.

d LPA: light intensity physical activity.

e SB: sedentary behavior.

f *P*<.05.

g PA: physical activity.

h Not applicable.

Adjusting for covariates may overfit models. Another set of unadjusted multilevel models was tested to determine correspondence between EMA-reported and device-based behaviors. Results did not differ substantially from the adjusted results presented in Table 3 regarding agreement between EMA-reported and device-based behaviors. Output from unadjusted multilevel models is in Multimedia Appendix 2.

### Acceptability

As indicated in Table 4, most participants (48/66, 72.7%) were satisfied with the study. More than half of the participants indicated that the length of the questionnaires was reasonable (ie, 37/66, 56.1% strongly disagreed or disagreed that the EMA questionnaires took too much time to complete). There were mixed responses regarding the convenience of the timing of EMA prompts (ie, 21/66, 31.8% agreed or strongly agreed prompts were delivered at a convenient time; 15/66, 22.7% disagreed or strongly disagreed). One participant noted in open-ended comments about the study: “Sometimes questionnaires were too early, or I was busy with my kids, and I couldn’t answer.” Another participant indicated a preference for more time before EMA prompts became inaccessible, with one participant noting that they would have liked to “get more than 30 minutes to answer [the EMA prompt].” Most participants indicated that the preferred number of EMA prompts per day was 3 (52/66, 78.8%) and study days was 7 (53/66, 80.3%). Though in open-ended responses, some participants reported the desire for EMA questionnaires to take less time to complete. Nearly half of the participants indicated that the accelerometer was not a nuisance to wear (29/66, 43.9%) or were neutral about wearing it (18/66, 27.3%), while 28.8% (19/66) indicated it was a nuisance to wear. More than four-fifths (56/66, 84.8%) of the sample indicated that they were satisfied with the compensation provided for completing the study.

**Table 4.** Participant feedback on study procedures (n=66^a^).

Item and response options	Values, n (%)
It took too much time to complete the questionnaires sent over the telephone.	
Strongly disagree	14 (21.2)
Disagree	23 (34.8)
Neutral	20 (30.3)
Agree	7 (10.6)
Strongly agree	1 (1.5)
Missing	1 (1.5)
The hours when the EMA^b^ prompts were sent over the telephone were convenient.	
Strongly disagree	6 (9.1)
Disagree	9 (13.6)
Neutral	30 (45.5)
Agree	18 (27.3)
Strongly agree	3 (4.5)
It was a nuisance wearing the activity monitor for 7 days.	
Strongly disagree	10 (15.2)
Disagree	19 (28.8)
Neutral	18 (27.3)
Agree	14 (21.2)
Strongly agree	5 (7.6)
This study demanded a lot of time.	
Strongly disagree	18 (27.3)
Disagree	26 (39.4)
Neutral	16 (24.2)
Agree	6 (9.1)
Strongly agree	0 (0)
I was satisfied with the compensation for this study.	
Strongly disagree	3 (4.5)
Disagree	0 (0)
Neutral	6 (9.1)
Agree	33 (50.0)
Strongly agree	23 (34.8)
Missing	1 (1.5)
Overall, what was your level of satisfaction with the study?	
Very unsatisfied	3 (4.5)
Unsatisfied	0 (0)
Neutral	14 (21.2)
Satisfied	33 (50.0)
Very satisfied	15 (22.7)
Missing	1 (1.5)
What is the maximum number of questionnaires you would be willing to complete on your mobile phone in a single day?	
3 prompts	52 (78.8)
4‐5 prompts	8 (12.1)
6‐8 prompts	6 (9.1)
What is the maximum number of consecutive days you would be willing to complete these study procedures?	
7 days	53 (80.3)
8‐11 days	7 (10.6)
12+ days	6 (9.1)

a One participant did not provide study feedback.

b EMA: ecological momentary assessment.

## Discussion

EMA can provide novel insights into the prediction and modeling of movement-related behaviors as it captures phenomena of interest in real-time and real-world environments. However, such a methodology is only useful and informative if participants are willing to comply with study procedures and the data collected through this method are valid. To our knowledge, this study is the first to investigate Latinas’ willingness, compliance, and accuracy in reports of behaviors in an EMA protocol designed to assess movement-related behaviors. We found that among a sample of emerging to midlife Latinas of Mexican or Mexican American origin and born outside of the United States, these women were willing to engage in and comply with the EMA research, demonstrated strong agreement in reporting engagement in PA and SB when compared to device-based measures, and, overall, had positive perceptions of the study procedures. This study provides encouraging findings for future EMA research with Latinas to better understand and intervene on movement-related behaviors.

Latinas demonstrated acceptable levels of compliance with the EMA protocol, answering approximately 72.5% (927/1279) and completing 69.7% (892/1279) of EMA prompts in this study. Across a variety of research designs, samples, and disciplines, the average compliance with an EMA protocol is approximately 79% [12]. Compared to EMA studies of movement behaviors exclusively among samples of women, compliance rates documented in this study were similar [27,28] or better than previous research [13]. Some of the PA EMA studies among women have implemented strategies to entice EMA compliance by offering additional compensation based on the percentage of EMA prompts answered [27,29]. However, Wrzus and Neubauer [12] found that compliance did not differ if EMA studies used incentives dependent on answering minimum percentage of prompts compared to incentives without compliance reinforcement. It appears the main compliance issue women in our study expressed was the convenience of EMA prompts (eg, timing). Further design considerations regarding EMA prompts among Latinas may be warranted.

Rates of activity monitor nonwear documented in this study were surprising, considering that participants generally reported that it was not a nuisance to wear the activity monitor. Commercial wearable devices may be more advantageous than research-grade devices as commercial devices tend to be water-resistant, comfortable to wear, and stylish, all of which can promote continuous wear with limited reasons for device removal. However, researchers should weigh the pros and cons of using commercially available devices for research purposes [30].

Our findings suggest that the likelihood of completing an EMA prompt increases significantly if Latinas are engaged in higher levels of SB and less LPA around the time of the prompt. Previous EMA studies have similarly documented that rates of compliance are improved if a participant is engaged in high levels of SB around the EMA prompt and that engagement in MVPA around the EMA was unrelated to compliance [16,17]. Other PA EMA studies have documented compliance differences based on concurrent engagement in MVPA (particularly driven by vigorous intensity PA) [15,31], but we did not observe such differences in compliance. This may be due to the relatively low levels of MVPA in our study sample. We did, however, find that engaging in more LPA around the prompt decreased the likelihood of completing an EMA prompt. Engaging in higher levels of LPA may reflect a general busyness of daily life among women, which may explain poorer compliance at those times.

One concern with EMA protocols is that they can potentially induce measurement reactivity (ie, changes in a construct because of intensively assessing it) [22,23]. Our finding that Latinas sat for significantly more time immediately following the EMA prompt as compared to immediately before the EMA prompt aligns with previous findings [15]. Of note, estimates in our study suggest that the difference in SB before and after the EMA prompt was approximately 30 seconds. This volume of change in SB may not have practical implications for Latinas’ health. Further, neither MVPA nor LPA significantly changed among Latinas because of completing the EMA prompt, aligning with previous work [15,23]. In general, findings from this study are promising as they suggest that completing an EMA prompt does not induce meaningful reactivity in PA or SB behaviors among Latinas.

Regarding the validity of EMA to assess PA and SB, on occasions when Latinas reported engaging in PA or SB via EMA, actigraphy-derived measures of those behaviors were greater than when those behaviors were not reported via EMA. Importantly, actigraph-derived MVPA increased by nearly 4 minutes when Latinas reported engaging in PA at the EMA prompt, but LPA only increased by about 2 minutes. This suggests that Latinas accurately differentiated between intensities of PA as they were instructed during the introductory training session and reminded at the beginning of each EMA prompt. Given the relatively low prevalence of MVPA in the ±15-minute window around the EMA prompt across all observations (mean 0.96, SD 2.87 minutes), the increase of nearly 4 minutes of device-based PA when PA was reported at the prompt indicates that participant reports were consistent with the behavioral engagement. The agreement between EMA-reported and device-based movement behaviors is consistent with findings from studies involving diverse samples of emerging, midlife, and older adults. These studies have shown increases of 2-4 minutes in device-based PA on occasions when PA was reported through EMA prompts, compared to occasions when it was not [15-17,32]. Ultimately, these findings indicate that Latinas accurately report their current movement behaviors through EMA.

Finally, the overall positive perceptions of the EMA study indicate that this type of smartphone-based study design is acceptable to assess movement behaviors in a sample of Latinas that have immigrated to the United States. Based on participants’ feedback, further refining ways to minimize study demands and improve the convenience of study procedures would be advantageous. Additional strategies such as customizing to participants’ sleep and wake time schedules or allowing more time to complete the EMA prompt could address issues of convenience. Lengthening the available window in which Latinas can respond to an EMA prompt may help limit potential burden. However, when deciding whether to lengthen the availability of the EMA questionnaire, researchers should weigh the potential benefits and costs of such a decision considering the phenomenon of interest they are interested in capturing. Finally, considerations around the number of items and time to complete at each EMA questionnaire may be necessary. Some EMA studies have reduced the number of items in EMA prompts, while also providing variety in the constructs assessed, by randomizing items so that only a portion of constructs are assessed at each EMA prompt [15,33]. Though such design considerations may only be applicable in studies with a larger number of EMA prompts within days or more days of assessment to ensure that all constructs are adequately captured to reflect the changing contexts of daily life.

Strengths of this study include the rigorous interrogation of factors that may influence potential feasibility and validity of a movement behavior EMA protocol among a socially vulnerable population of Latinas. However, study limitations should be noted. Although simulation studies indicate that in intensive longitudinal study samples of more than 50 participants with assessments within participants can generate reliable fixed and random estimates as well as standard errors [26], our sample size was relatively small (N=67), which may limit the generalizability of findings. Additionally, we obtained a larger percentage of Latinas meeting PA guidelines compared to national averages. Further, most of our sample identified as Mexican or Mexican American and was born outside of the United States. There may be important differences in terms of feasibility, validity, and acceptability depending on Hispanic origin and nativity status. Relatedly, we did not take height and weight measurements to determine participants’ Body Mass Index (BMI). Some studies have indicated differences in EMA compliance by BMI [15]. Examining differences in compliance by BMI is an important direction for future EMA research among Latinas. With respect to our study design, because a random, signal-contingent prompting schedule was used, it is likely that not all bouts of PA and SB were captured. Further, EMA items did not assess the duration or intensity of PA or SB.

The findings from our study indicate that EMA methods are a feasible, valid, and acceptable approach to use with Latinas to understand PA and SB. Although some strategies may need to be implemented in future work to enhance compliance with smartphone and accelerometer-based study procedures, our study provides timely, new evidence that EMA can be effectively used among Latinas to capture movement behaviors. Latinas represent a vulnerable and understudied population in PA research. Using EMA in such a group can shed new light on the antecedents and consequences of movement behaviors, leading to new intervention targets delivered in the context of Latinas’ daily lives. The findings from this study represent an essential first step in that process.

## Supplementary material

10.2196/75855 Multimedia Appendix 1 Ecological momentary assessment (EMA) screenshots.

10.2196/75855 Multimedia Appendix 2 Predicting minutes of movement behaviors in the 30-minute window (±15 minutes) around the EMA prompt unadjusted for covariates to determine correspondence between EMA-reported and device-based behaviors. EMA: ecological momentary assessment.

## Abbreviations

EMA: ecological momentary assessment
LPA: light-intensity physical activity
MVPA: moderate-to-vigorous-intensity physical activity
OR: odds ratio
PA: physical activity
SB: sedentary behavior
