# Supplementary material for: Capturing Movement Behaviors in Latinas: Feasibility, Validity, and Acceptability Study of an Ecological Momentary Assessment Protocol
Source: JMIR Hum Factors. 2025 Nov 5;12:e75855. doi: 10.2196/75855 (PMC12588591; doi:10.2196/75855)
Supplement: Multimedia Appendix 1 [file humanfactors-v12-e75855-s001.docx]

**Figure S1.** Ecological momentary assessment (EMA) screenshots.

| Screen 1  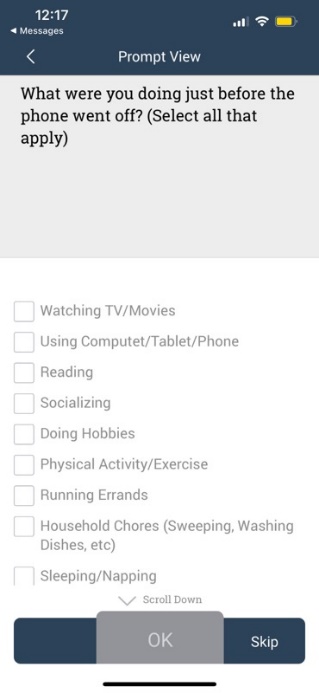 | Screen 2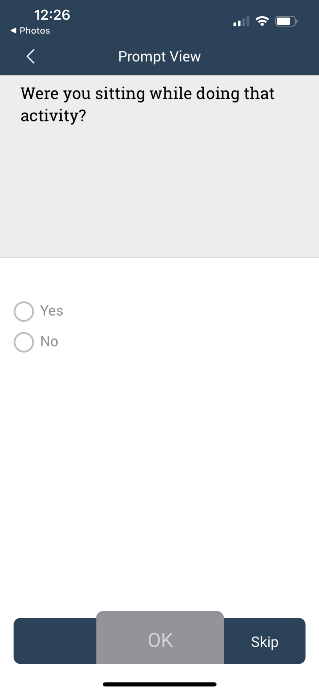 | Screen 3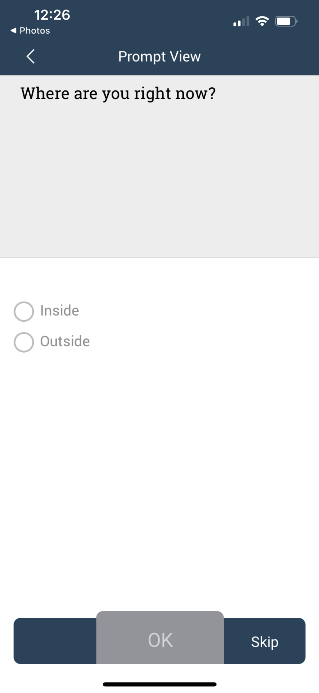 | Screen 4  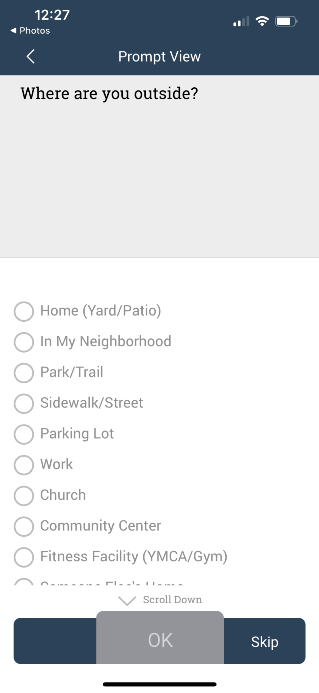 |
| --- | --- | --- | --- |
| Screen 5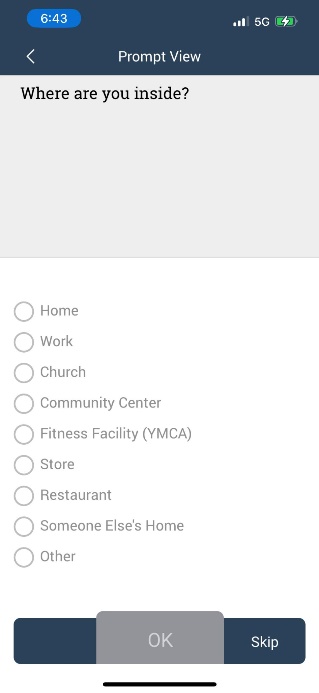 | Screen 6  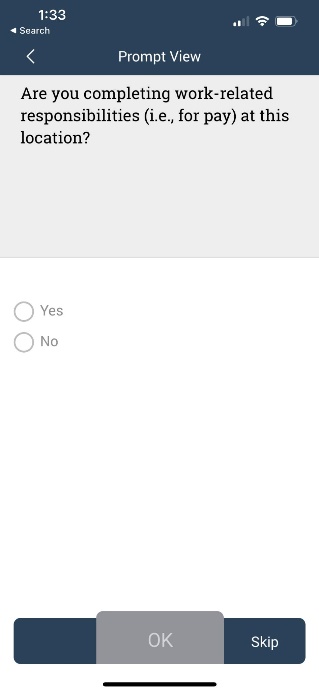 | Screen 7  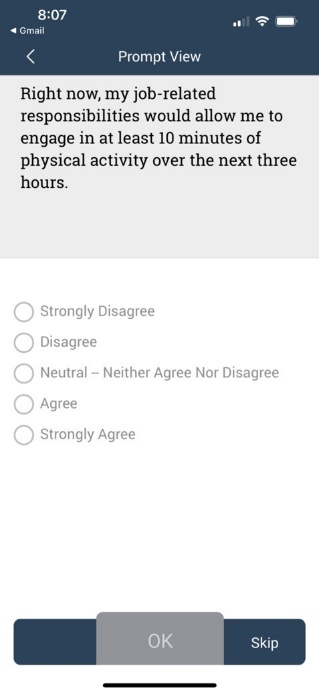 | Screen 8 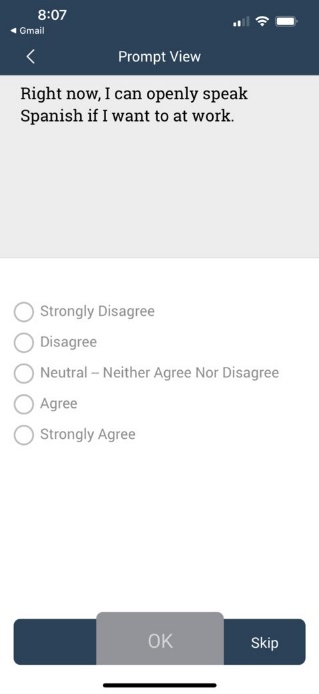 |

| Screen 9  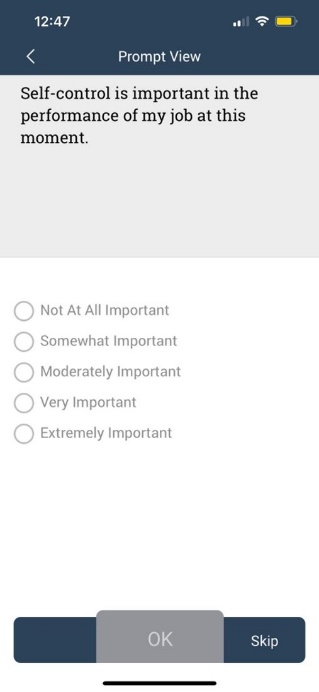 | Screen 10  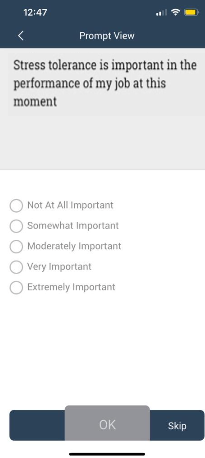 | Screen 11  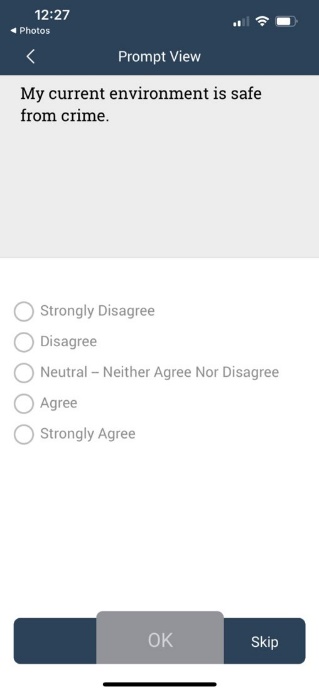 | Screen 12  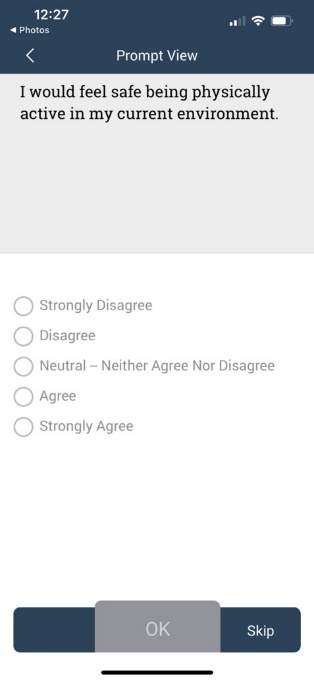 |
| --- | --- | --- | --- |
| Screen 13  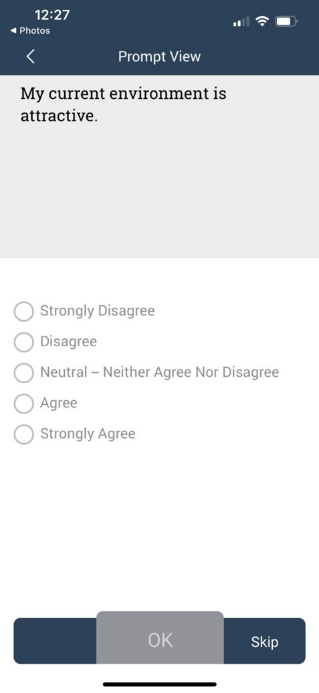 | Screen 14  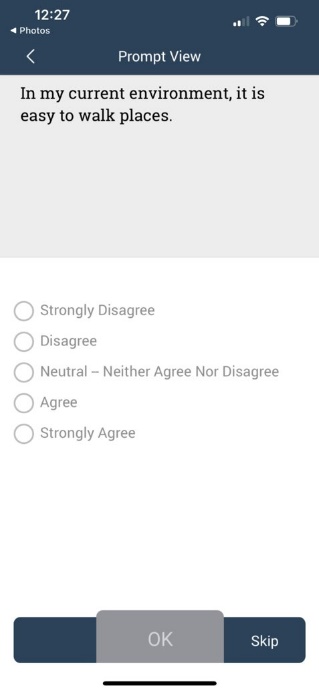 | Screen 15  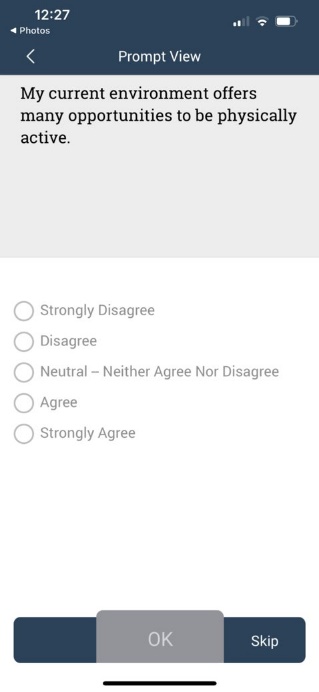 | Screen 16 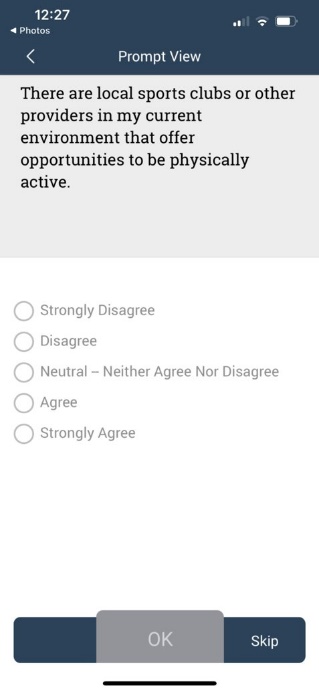 |
| Screen 17  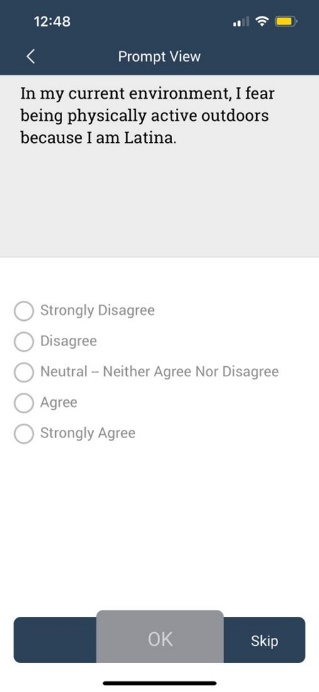 | Screen 18  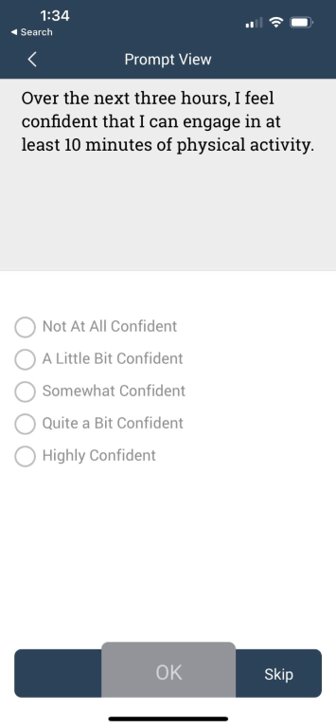 | Screen 19  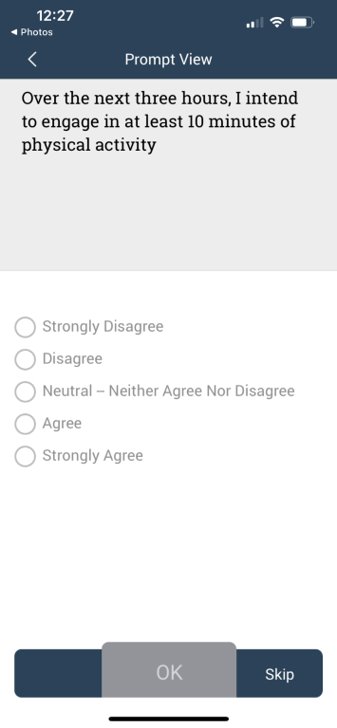 | Screen 20 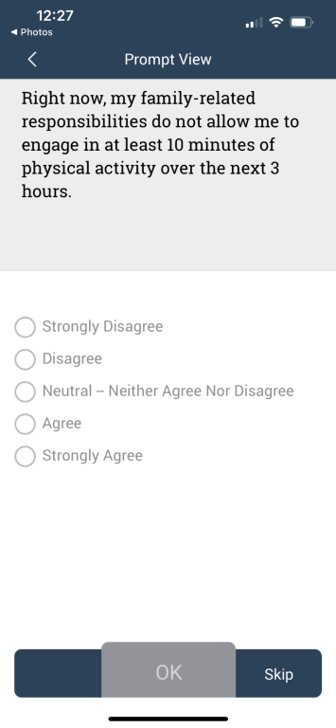 |
| Screen 21  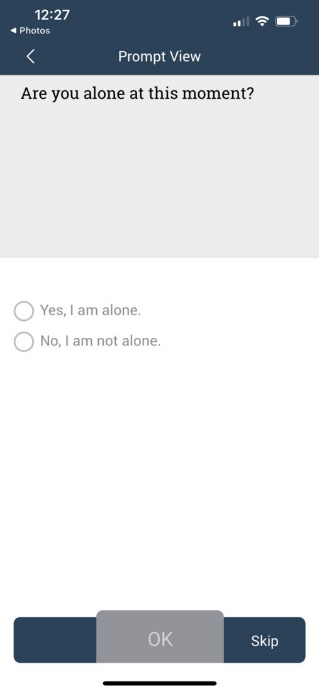 | Screen 22  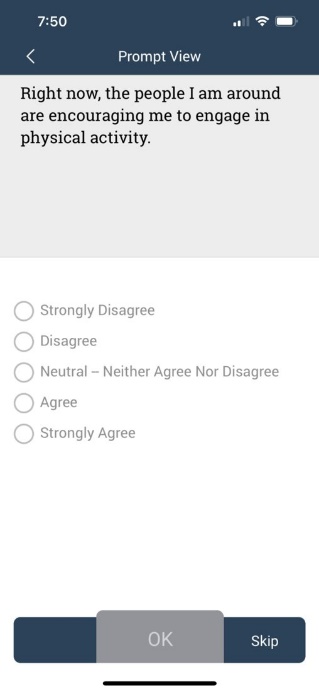 | Screen 23  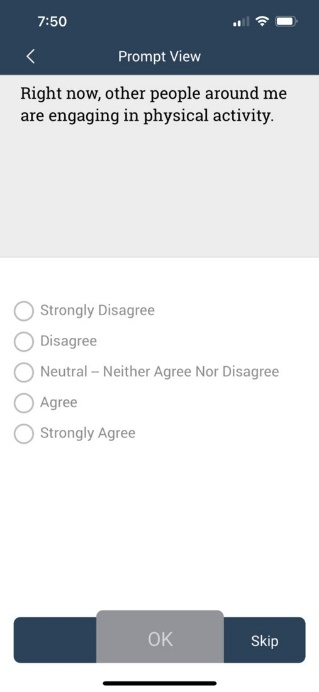 | Screen 24 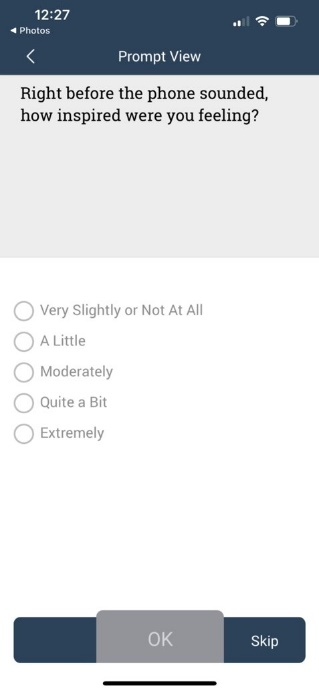 |
| Screen 25 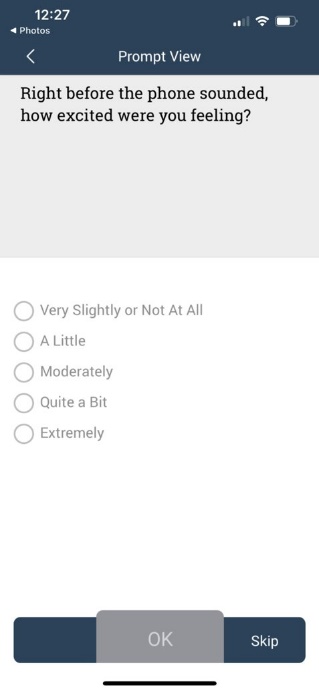 | Screen 26  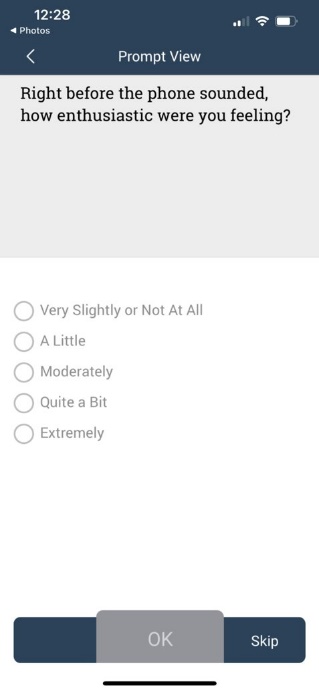 | Screen 27  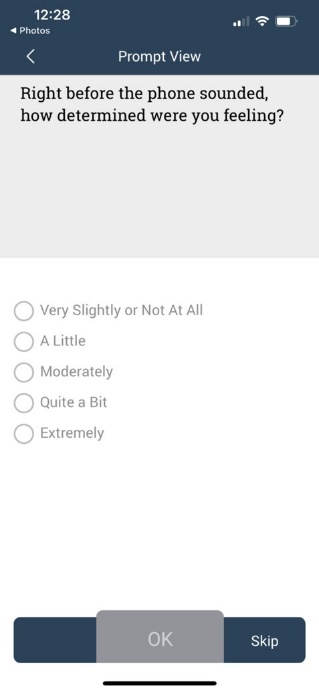 | Screen 28  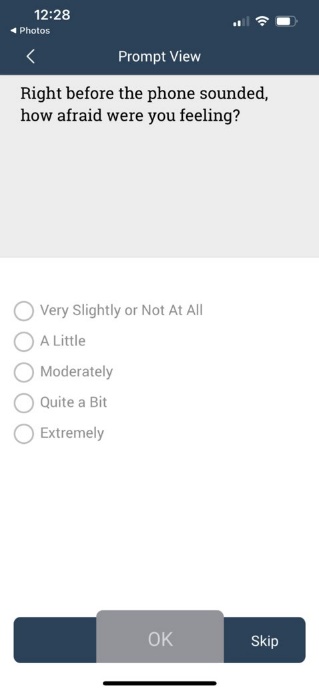 |
| Screen 29  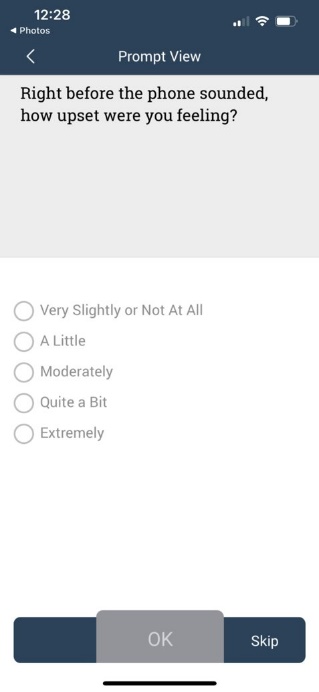 | Screen 30  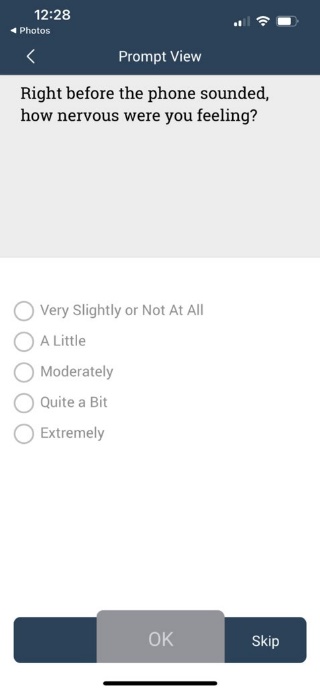 | Screen 31  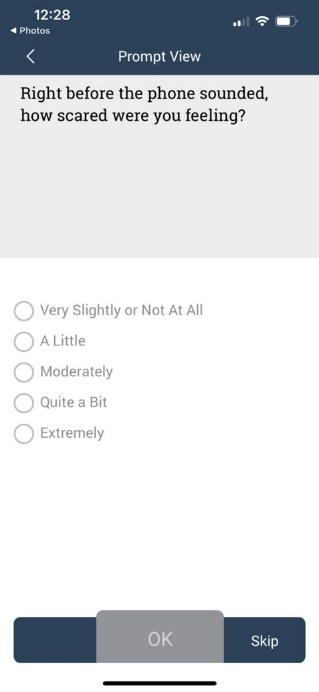 | Screen 32  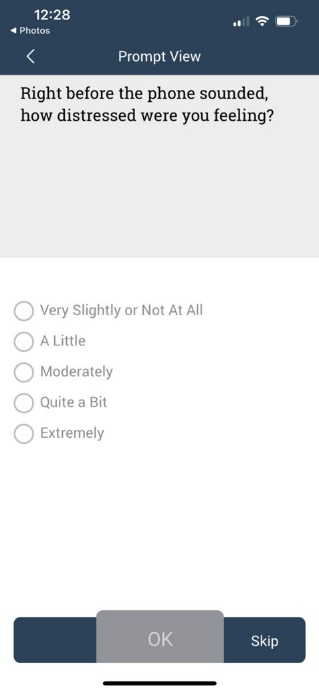 |
| Screen 33  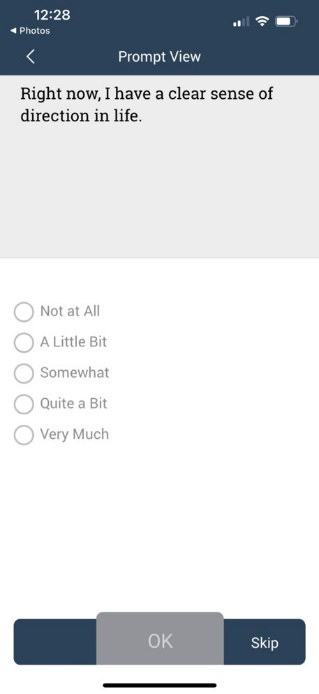 |  |  |  |

Note. If participant responded “Outside” to screen 3, they were directed to screen 4. If participant responded “Inside” to screen 3, they were directed to screen 5. If participant responded “Yes” to Screen 6, they were directed to Screens 7-10. If participant responded “No, I am not alone” to screen 21, they were directed to screens 22-23.
