# Supplementary material for: Capturing Movement Behaviors in Latinas: Feasibility, Validity, and Acceptability Study of an Ecological Momentary Assessment Protocol
Source: JMIR Hum Factors. 2025 Nov 5;12:e75855. doi: 10.2196/75855 (PMC12588591; doi:10.2196/75855)
Supplement: Multimedia Appendix 2 [file humanfactors-v12-e75855-s002.docx]

**Table S1.** Predicting minutes of movement behaviors in the 30-minute window (±15 minutes) around the EMA prompt unadjusted for covariates to determine correspondence between EMA-reported and device-based behaviors.

|  | Multilevel linear regression with MVPA as outcome | Multilevel linear regression with LPA as outcome | Multilevel linear regression with SB as outcome |
| --- | --- | --- | --- |
|  | Unstandardized Coefficient (SE) | Unstandardized Coefficient (SE) | Unstandardized Coefficient (SE) |
| Fixed Effects |  |  |  |
| Intercept | 0.65* (0.09) | 9.59* (0.36) | 16.84* (0.47) |
| EMA-Reported PA | 3.60* (0.43) | 2.35* (1.18) | -- |
| EMA-Reported SB | -- | -- | 4.31* (0.52) |
| Random Effects |  |  |  |
| Intercept | 0.06 (0.08) | 5.09 (1.59) | 4.91 (1.64) |
| Residual | 4.92 (0.28) | 34.84 (2.03) | 40.92 (2.37) |

Note: * *P* < 0.05. – not applicable. All models were based on 66 participants with 657 observations for models predicting MVPA and LPA and 656 observations for the model predicting SB.
